# Interdisciplinary approach to pediatric rehabilitation - The MDT method

**DOI:** 10.1192/j.eurpsy.2022.1938

**Published:** 2022-09-01

**Authors:** E. Vashdi

**Affiliations:** Yaelcenter institute, Clinic, Aloney Aba, Israel

**Keywords:** Interdisciplinary, MDT, autism, rehabilitation

## Abstract

**Introduction:**

Interdisciplinarity involves the combining of two or more academic disciplines into one activity (e.g. a research project). It is about creating something new by crossing traditional boundaries between academic disciplines or schools of thought, as new needs and professions emerge, and thinking across them. The roots of interdisciplinarity can be found hundreds of years ago however, In the twentieth century it became a more academic movement.

**Objectives:**

Interdisciplinarity also involves integrated thinking, borrowing ideas and metaphors, collaboration, flexibility in thinking, innovation and complex problem solving. The traditional professional model is usually disciplinary. Complex problems sometimes demand interdisciplinary solution. There is a constant dispute between the disciplinary and interdisciplinary approaches.

**Methods:**

The MDT (Multidimessional therapy) method is part of the transdisciplinary approach. This is the next step after interdisciplinarity in which there are no boundaries between disciplines and the role of a primary therapist is established.

**Results:**

The MDT is a system that implements the integrative therapy perception, and strives for optimal integration of all developmental areas, disciplines, methods, goals, schools, activities etc. So the child is seen as a whole and all developmental areas are considered in unison. The integration takes place at different aspects of therapy: evaluation, analysis process, case management, team work, disciplines and methods, therapist, goals and exercises.The MDT uses variuos integrative tools evaluation such as analysis process, primary therapist or integrative goals and exercises.

**Conclusions:**

This lecture will present the structure and uniqueness of the system and the approach in order to introduce the beneficiary aspects of the integrative model in treatment of complex cases.

**Disclosure:**

No significant relationships.

